# Monitoring Hepatic Biomarker Responses in Caged *Oreochromis niloticus* Under Chronic Exposure to Micropollutants in the Iguaçu River

**DOI:** 10.3390/jox16050162

**Published:** 2026-08-27

**Authors:** Lorena Bavia, Rayanne Seibel Littig, Manuela Santos Santana, Milena Carvalho Carneiro, Luiza Santos Barreto, Thaís Muniz Vasconcelos, Marco Antonio Ferreira Randi, Cesar Castro Martins, Andrea Pinto De Oliveira, Iracema Opuskevitch, Fernando Cesar Alves Da Silva Ferreira, Juan Esquivel-Muelbert, Ciro Alberto De Oliveira Ribeiro, Maritana Mela Prodocimo

**Affiliations:** 1 Laboratório de Toxicologia Celular, Departamento de Biologia Celular, Universidade Federal do Paraná, Curitiba CEP 81531-970, PR, Brazil; 2 Faculdades Pequeno Príncipe, Av. Iguaçu, 333, Rebouças, Curitiba CEP 80230-020, PR, Brazil; 3 Instituto de Pesquisa Pelé Pequeno Príncipe, Av. Silva Jardim, 1632, Água Verde, Curitiba CEP 80250-060, PR, Brazil; 4 Laboratório de Ecologia Marinha, Centro de Estudos do Mar, Universidade Federal do Paraná, Pontal do Paraná CEP 83255-000, PR, Brazil; 5 Departamento de Imunologia, Instituto de Ciências Biomédicas, Universidade de São Paulo, São Paulo CEP 05508-000, SP, Brazil; 6 Instituto Oceanográfico, Universidade de São Paulo, São Paulo CEP 05508-120, SP, Brazil; 7 Departamento de Química, Universidade Federal do Paraná, Curitiba CEP 81531-980, PR, Brazil; 8 Copel GeT-SOS/DNGT, José Izidoro Biazetto, 185, Bloco a, Curitiba CEP 81200-240, PR, Brazil; 9 School of Natural Sciences, Macquarie University, Sydney, NSW 2109, Australia

**Keywords:** Hepatotoxicity, biomonitoring, water quality, freshwater fish, micropollutants

## Abstract

Chemical pollution from industrial, agricultural, and urban activities represents a major threat to freshwater ecosystems and aquatic organisms. This study evaluated hepatic biomarker responses in *Oreochromis niloticus* (Nile tilapia) maintained under chronic environmental exposure to water from the Iguaçu River, one of the most polluted urban rivers in Brazil. Juvenile fish were kept in cages at three sites along the river, and hepatic biomarkers were assessed after 15 and 22 months of environmental exposure. Fish showed severe histopathological liver lesions, activation of antioxidant defenses, and oxidative stress responses accompanied by increased DNA damage. Immunological responses, particularly melanomacrophage proliferation and granuloma formation, were also observed across the monitored exposure scenarios. Alterations in plasma biochemical parameters, including AST, ALT, LDH, albumin, and globulin, were consistent with changes in hepatic function. Overall, the integrated biomarker responses revealed distinct patterns of biological alteration among the monitored exposure scenarios and were consistent with chronic exposure to complex environmental contaminant mixtures. These findings are consistent with alterations in liver integrity in fish maintained under long-term environmental exposure in the Iguaçu River. The study highlights the sensitivity of Nile tilapia as a bioindicator species for aquatic biomonitoring and provides valuable information to support environmental monitoring, risk assessment, and conservation strategies for the Iguaçu River Basin.

## 1. Introduction

The contamination of aquatic environments worldwide with micropollutants has become a matter of great concern due to their potential negative effects on aquatic biodiversity and human health [1,2,3,4]. These chemical compounds pose considerable toxicological threats to fish species, particularly when they are chronically exposed, or when they are present in complex chemical mixtures. Chronic exposure to a combination of pollutants can induce a series of physiological changes that can result in oxidative stress and cause damage at various levels of biological organization, including molecules, cells, tissues, and organs [5,6]. Ultimately, these effects can threaten fish populations by reducing reproductive success [7]. Some of these chemicals can also bioaccumulate and, in some cases, biomagnify throughout trophic chains, impacting human populations that consume contaminated fishery resources [8,9].

Evaluating the impact of micropollutants in aquatic systems is challenging and requires analytical and modeling tools, along with biological indicators, to assess their presence, distribution, and bioavailability, as well as associated biological effects [2,10]. Living organisms have developed physiological mechanisms to cope with chemical exposure, primarily through metabolism, also known as biotransformation [11]. In fish, this process takes place mostly in the liver, an integral component of the digestive system. The liver regulates the metabolism of carbohydrates, proteins, and lipids, and is also paramount in the storage, metabolization and excretion of hormones and a variety of other essential chemicals [8,12,13]. Upon exposure to contaminants, a metabolic decline occurs in fish, as hepatic nutrient metabolism is altered [10,14,15]. Consequently, the liver is considered the most suitable organ in fish to support biomonitoring, as it integrates physiological and biochemical functions that, when altered, may produce diverse measurable biomarkers of prior exposure to pollutants [3,8].

The Iguaçu River basin is the largest freshwater body in the state of Paraná, and is recognized for its natural beauty and for its socio-economic and environmental relevance [3,4,16,17]. However, due to intense anthropogenic activity along its course, the Iguaçu River is currently classified as the second most polluted urban river in Brazil [2,4,18,19]. The Iguaçu River also harbours a high rate of endemic ichthyofauna [20], which makes the region a priority area for the conservation and sustainable use of Brazilian biodiversity [21,22]. In our previous research, we underscored how the deteriorating water conditions in the Segredo Hydroelectric Reservoir impact Nile tilapia (*Oreochromis niloticus*) across different life stages [20,22]. However, most studies to date have assessed the effects of chemical exposure over short periods, and consequently, the impacts of chronic exposure to chemical contaminants remain insufficiently understood. The present study expands upon previous investigations by incorporating multiple sampling sites and extended exposure periods (15 and 22 months), providing additional insights into the biological responses of aquatic organisms to chronic exposure to complex mixtures of environmental contaminants.

Active biomonitoring using caged sentinel species provides accurate insights into contaminant bioavailability, bioaccumulation, and associated biological effects [23,24,25,26]. The Nile tilapia was chosen because previous studies have demonstrated that this species exhibits rapid and sensitive responses to a wide range of pollutants in both laboratory and field conditions [3,27]. Moreover, this species is extensively cultivated and holds great economic importance in southern Brazil. It is also widely used in ecotoxicological research worldwide, enabling meaningful comparisons across studies [3,4].

Here, we used an active biomonitoring approach to compare biomarker responses in caged Nile tilapia among sampling locations representing different environmental exposure scenarios within the Segredo Reservoir. Hepatic biomarkers were selected to provide an integrated assessment of biochemical responses, oxidative stress, genotoxicity, and histopathological alterations associated with chronic environmental exposure. We hypothesized that chronic exposure to water under different environmental exposure scenarios in the Iguaçu River would be associated with distinct hepatic biomarker patterns, including histopathological alterations and oxidative stress responses, followed by genotoxic responses and changes in plasma biochemical parameters consistent with altered hepatic function. Furthermore, we expected that prolonged exposure would reveal biological responses not readily detected by conventional water-quality assessments, highlighting the value of hepatic biomarkers as sensitive tools for environmental monitoring.

## 2. Materials and Methods

### 2.1. Study Site

**Figure 1:**
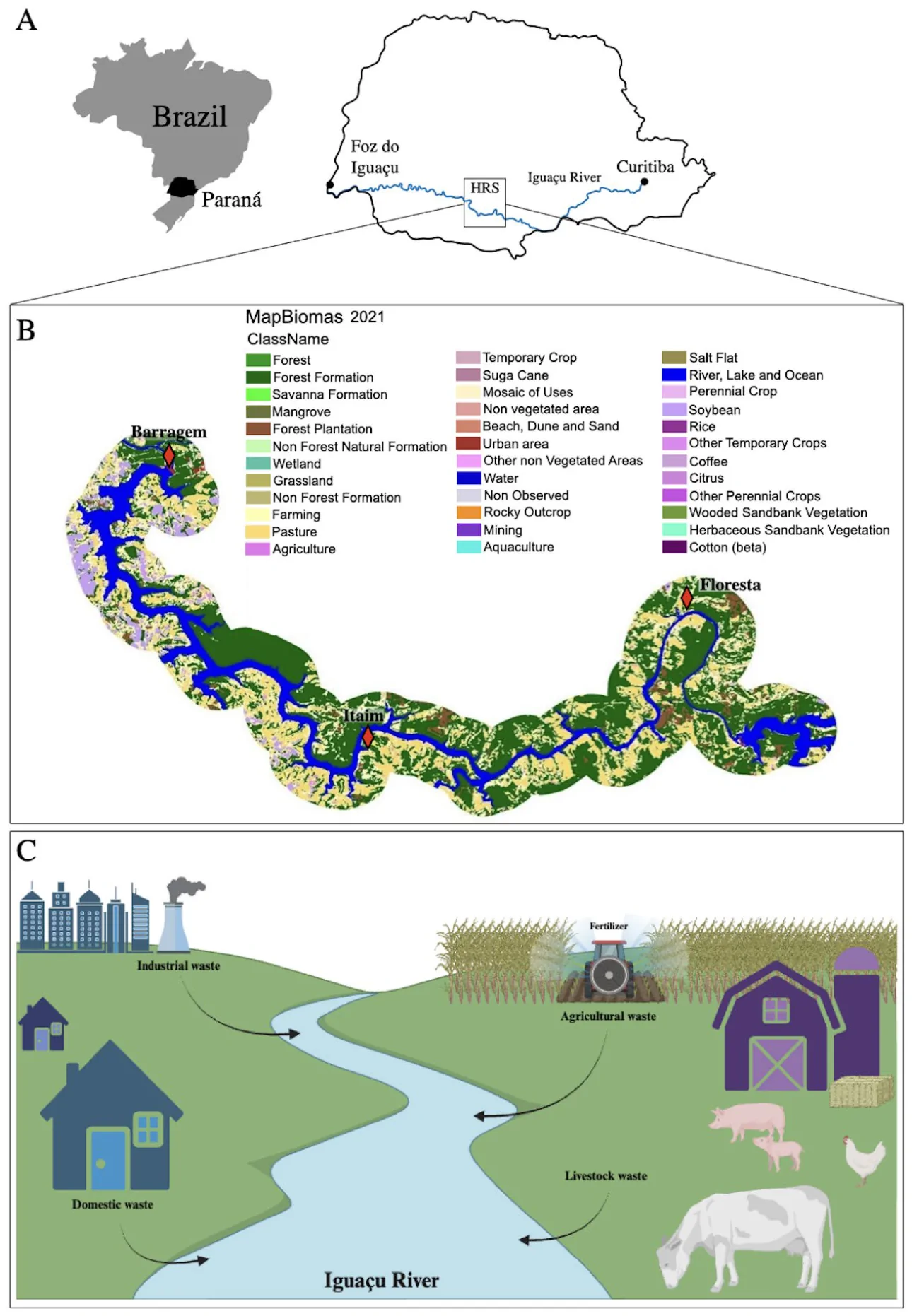
Locations of selected sampling sites along the Iguaçu River. (**A**) The maps show the state of Paraná in Brazil, the course of the Iguaçu River originating from the capital city, Curitiba, and the specific location of the study area indicated by Hydroelectric Reservoir of Segredo (HRS). The geographical names shown in Portuguese (e.g., Paraná, Curitiba, and Foz do Iguaçu) are official names of Brazilian state and cities and are therefore retained in their original form. (**B**) The map shows the different studied exposure scenarios in the reservoir and the distribution of land use within a 3 km radius of the river body in 2021. (**C**) The scheme shows anthropogenic activities in the Iguaçu River.

The Iguaçu River originates in the metropolitan region of Curitiba, Brazil, and flows for 1320 km until it joins the Paraná River at the Iguaçu Falls. The river is a critical water source for approximately 4.5 million people in southern Brazil, supplying drinking water, supporting irrigation, and contributing to hydroelectric energy production [28]. The Hydroelectric Reservoir of Segredo (HRS) (Figure 1) is located 455 km downstream from the metropolitan region of Curitiba and retains the chemical imprint of several human activities. This reservoir was selected because previous studies have demonstrated the presence of environmental pollution [1,3,4,13,17,23]. Thus, three sites with distinct environmental contexts were chosen to investigate the influence of human activities within the HSR. Because the entire Segredo Reservoir is influenced by different degrees of anthropogenic activities, no truly uncontaminated reference site was available within the same environmental system. Therefore, the study was designed to compare biological responses among in situ exposure scenarios representing distinct environmental exposure conditions rather than against a pristine environmental baseline.

- *Barragem* (BA) (25°47′28″ S, 52°07′08″ W): an area influenced by surrounding urban and agricultural activities;
- *Iratim* (IR) (26°00′10″ S, 51°57′56″ W): a site with comparatively low direct human influence;
- *Floresta* (FL) (25°55′01″ S, 51°42′40″ W): a site characterized by surrounding urban and industrial activities.

### 2.2. Experimental Caging

Juveniles *O. niloticus* (*n* = 180, weighing 10–20 g) were obtained from a commercial fish hatchery (Piscicultura Panamá) located in Paulo Lopes, Santa Catarina, Brazil, (27°57′35″ S; 48°45’28″ W). Fish were transported to exposure scenarios in plastic bags filled with 10 L of water and oxygen. At each of the three exposure scenarios, fish were distributed into two 8 m^3^ plastic mesh cages, with 30 fish per cage. The cages were constructed with 20 mm non-toxic plastic mesh, allowing for free water circulation, and were fully submerged near the sediment and firmly anchored to prevent displacement. In this active biomonitoring study, the cages served exclusively as standardized containment structures to maintain sentinel fish under natural in situ exposure conditions and were not considered independently manipulated experimental systems. One cage at each site was sampled after 15 months of exposure, and the second cage at each site was sampled after 22 months. Thus, each cage corresponded to a single monitored exposure scenario defined by sampling site and exposure duration. At each sampling time, all fish from the respective cage (*n* = 30) were collected for biomarker analyses. Biomarker measurements were performed on individual fish, which constituted the observational units. Fish within each cage were considered biological subsamples sharing a common exposure environment. Accordingly, the results describe individual-level variation and patterns among the exposure scenarios rather than independently replicated site or exposure-duration effects. During this period, the fish were fed commercial pellets (Supra Juvenil^®^ and Aqua Fish^®^, Alisul Alimentos S/A, São Leopoldo, Rio Grande do Sul, Brazil; 32% protein) three times per week.

### 2.3. Fish Sampling

Following the in situ exposure scenario, the fish were retrieved and rapidly transported (in river water with aeration) to the Experimental Laboratory of the Companhia Paranaense de Energia (COPEL), Curitiba, Paraná, Brazil, located near the sampling station. Prior to blood sampling and dissection, the fish were anesthetized with MS-222 (tricaine methanesulfonate, 0.02% in water), and the body weight (in g) and total length (in cm) were recorded for each individual. Blood was collected from the caudal vessel immediately after anesthesia, and plasma was separated via centrifugation. Subsequently, the individuals were euthanized by cervical section, and then dissected for identification. Liver samples were either stored at −80 °C for future chemical and biochemical analyses, or fixed in ALFAC solution containing 70% ethanol (Merck, Darmstadt, Germany), formaldehyde, and acetic acid (Synth, Diadema, São Paulo, Brazil) for optical microscopy. Due to the field design, site allocation was known during field procedures; however, all samples were coded prior to laboratory analyses. Biochemical, genotoxic, and histopathological evaluations were performed under blinded conditions, and the identity of the sampling location was revealed only after completion of all analyses.

### 2.4. Somatic Characteristics

The body weight and total length of the fish were obtained to determine Fulton’s condition factor (K), calculated using the formula: K = (W/L^3^) × 100, where W is the body and L is the total length (cm) of individual fish [29]. The liver weight was measured to determine the hepatosomatic index (HSI), using the formula: HSI = (W*_Liver_*/W*_Fish_*) × 100, where W*_Liver_* represents the liver weight and W*_Fish_* the weight of the fish [30].

### 2.5. Water, Sediment and Tissue Sampling

Water samples were collected from each exposure scenario at a depth of 30 cm below the water surface, refrigerated, and transported to the Federal University of Paraná, following the procedure described by [4]. Water samples intended for organic compound analysis were stored in amber glass bottles and kept under refrigeration at 4 °C until analysis, while those water samples intended for major and trace element analysis were acidified with nitric acid (pH 2) prior to storage. Sediment was sampled from a depth of 5 m using a Van Veen sediment sampler and stored in polypropylene flasks or aluminum containers for metal and organic compound analyses, respectively, and maintained at −20 °C until analysis. The muscle samples were collected and stored at −20 °C until analysis.

### 2.6. Chemical Analysis

Organic contaminant analyses were performed at the Laboratory of Organic Geochemistry and Marine Pollution (LaGPoM) at the Center for Marine Studies (CEM) at the Federal University of Paraná, Pontal do Paraná, Brazil. These analyses were conducted on water, sediment, and tissue (muscle) samples to determine the concentration of polycyclic aromatic hydrocarbons (PAHs), organochlorine pesticides (OCPs), polybrominated diphenyl ethers (PBDEs), and polychlorinated biphenyls (PCBs). The extraction of organic contaminants from water followed the EPA method 3510C (Separatory Funnel Liquid–Liquid Extraction, US EPA, 1996), as described by [31]. Sediment extraction was based on the protocol described by [32], whereas tissue extraction followed the protocol described by [33] with minor modifications. Metal analyses were conducted at the Analytical Center of the Department of Chemistry at the Federal University of Paraná, utilizing both water and sediment samples. The trace elements such as cadmium (Cd), lead (Pb), copper (Cu) zinc (Zn) and arsenic (As) and major metals aluminum (Al), iron (Fe) and manganese (Mn), were considered due to the historical and recent analyses [3,20]. For the quantification of the metals, Cu, Zn, Al, Fe and Mn were quantified by inductively coupled plasma optical emission spectrometry (ICP OES) and Cd, Pb, and As were quantified by graphite furnace atomic absorption spectrometry (GFAAS), following the procedure used by [4].

### 2.7. Oxidative Stress Parameters and Oxidative Damage

Liver samples were homogenized in a phosphate buffer (0.1 mol L^−1^, pH 7.0), and centrifuged at 15,000 *g* for 30 min, at 4 °C. Total protein concentration was determined according to [34], utilizing bovine serum albumin (BSA) to establish the standard curve. Catalase (CAT) activity was measured at 240 nm based on the [35] protocol; Superoxide dismutase (SOD) activity was measured at 440 nm according to [36]; Glutathione-S Transferase (GST) activity was measured at 340 nm according to [37], and Lipid peroxidation analysis (LPO) was carried out using the ferrous oxidation xylenol orange assay with absorbance measured at 570 nm according to [38]. Finally, the concentration of non-protein thiols (NPT) was determined following [39]. All biomarker readings were performed using a Varioskan LUX multimode microplate reader (Thermo Fisher Scientific, Waltham, MA, USA).

### 2.8. Serum Biochemical Parameters

Serum samples were assayed for alanine aminotransferase (ALT; cat. K035), aspartate aminotransferase (AST; cat. K034), alkaline phosphatase (ALP; cat. K224), albumin (cat. K040), total protein (cat. K031), lactate dehydrogenase (LDH; cat. K014), γ-glutamyl transferase (GGT; cat. K080), using commercially available enzymatic assay kits (Bioclin Quibasa, Belo Horizonte, MG, Brazil) following the manufacturer‘s instructions. Biocontrol N (human normal levels; cat. K073) and Biocontrol P (human pathological levels; cat. K074) from Bioclin Quibasa were used as internal controls in all biochemical assays, as previously described by [40]. In addition, globulin levels were calculated using the following formula: globulin (in g dL^−1^) = total protein (in g dL^−1^) − albumin (g dL^−1^) [40,41].

### 2.9. Histopathological Procedures

Liver samples were fixed in an ALFAC solution for 16 h at room temperature. The tissues were embedded in paraffin, sectioned at 5 μm, stained with hematoxylin and eosin, and examined under a Zeiss^®^ Axiophot photomicroscope, (Carl Zeiss, Oberkochen, Germany). Histopathological evaluation followed a semiquantitative approach based on the method described by [42]. Hepatic histopathological alterations were determined based on lesion severity. Melanomacrophage centers (MMC) were analyzed following the protocol by [43]. A detailed description of the histological procedures is available in the Supplementary Materials (Methods S1).

### 2.10. Genotoxicity

DNA strand breaks were measured in liver tissue using an alkaline precipitation assay [44], which is based on the potassium dodecyl sulfate precipitation of protein-bound genomic DNA. The precipitated DNA strands were quantified using fluorescence (λ_ex_ 360 nm and λ_em_ 450 nm) after staining. Standard solutions of salmon sperm DNA were used for calibration. Protein concentrations were determined spectrophotometrically at 595 nm [34], with BSA as the standard. All biomarker analyses were performed using a microplate reader (BioTek Synergy™ HT, Biotek Instruments, Winooski, VT, USA).

### 2.11. Statistical Analysis

Normality and homogeneity of variances were verified through Shapiro–Wilk and Bartlett tests, respectively. Additionally, diagnostic plots were visually inspected to ensure assumptions were met. Whenever necessary, data were transformed using square root or logarithmic transformations. Individual fish were considered observational units for biomarker measurements because the objective of the study was to characterize biological responses among organisms exposed under each environmental scenario. Cages served as standardized containment structures for in situ exposure rather than as independently manipulated experimental units, as proposed by [45]. Since only one cage was deployed per site × exposure-time combination, cage-level replication was not available. Therefore, fish within each cage were considered biological subsamples sharing a common exposure environment, and the statistical analyses were interpreted as describing individual-level variation within and among the monitored exposure scenarios. Accordingly, *p*-values obtained from these analyses are conditional on the observed cages and should not be interpreted as evidence of independently replicated differences among the six sampled exposure scenarios. Two–way ANOVAs were used as exploratory analyses to characterize individual-level variation among the six sampled exposure scenarios defined by the combination of sampling site (*Barragem*, *Floresta*, and *Iratim*) and exposure duration (15 and 22 months). Therefore, significant ANOVA results should not be interpreted as an overall effect of site and/or exposure periods in biomarker responses, but as mean differences in sampled cages of exposed fish. For exploratory comparison significance criterion (*p* < 0.05), means were compared using the Scott–Knott (SK) a posteriori test, which is a reliable clustering procedure for multiple comparisons [46]. The Scott–Knott algorithm is advantageous because it is not affected by the overlapping problems found in tests such as Tukey, Duncan, and Student–Newman–Keuls (SNK), which may classify treatment levels into different groups simultaneously. Because the melanomacrophage center (MMC) variable did not meet the normality assumption, it was analyzed using a generalized linear model (GLM)with a quasi-Poisson distribution. Post hoc comparisons among the sampled exposure scenarios were evaluated using least-squares means (lsmeans function in emmeans R package). For both ANOVA and GLM models, effect size measures (⍵^2^) were calculated as a proportion of variance explained in the response variable by each of the model’s terms (i.e., Site, Time and their interaction).

A principal component analysis (PCA) was performed to characterize multivariate patterns among biochemical biomarkers, somatic indexes, histopathological responses and the sampled exposure scenarios. To this end, variables were checked for normality, outliers and collinearity, and then standardized. Data analysis and graphs were generated using R programming language, using Scott-Knott and sciplot packages [47,48,49]. The PCA was used as an exploratory analysis to visualize multivariate patterns in biomarker responses across the six sampled exposure scenarios and exposure durations, providing a complementary description of the integrated biological responses.

## 3. Results

### 3.1. Somatic Indexes

**Figure 2:**
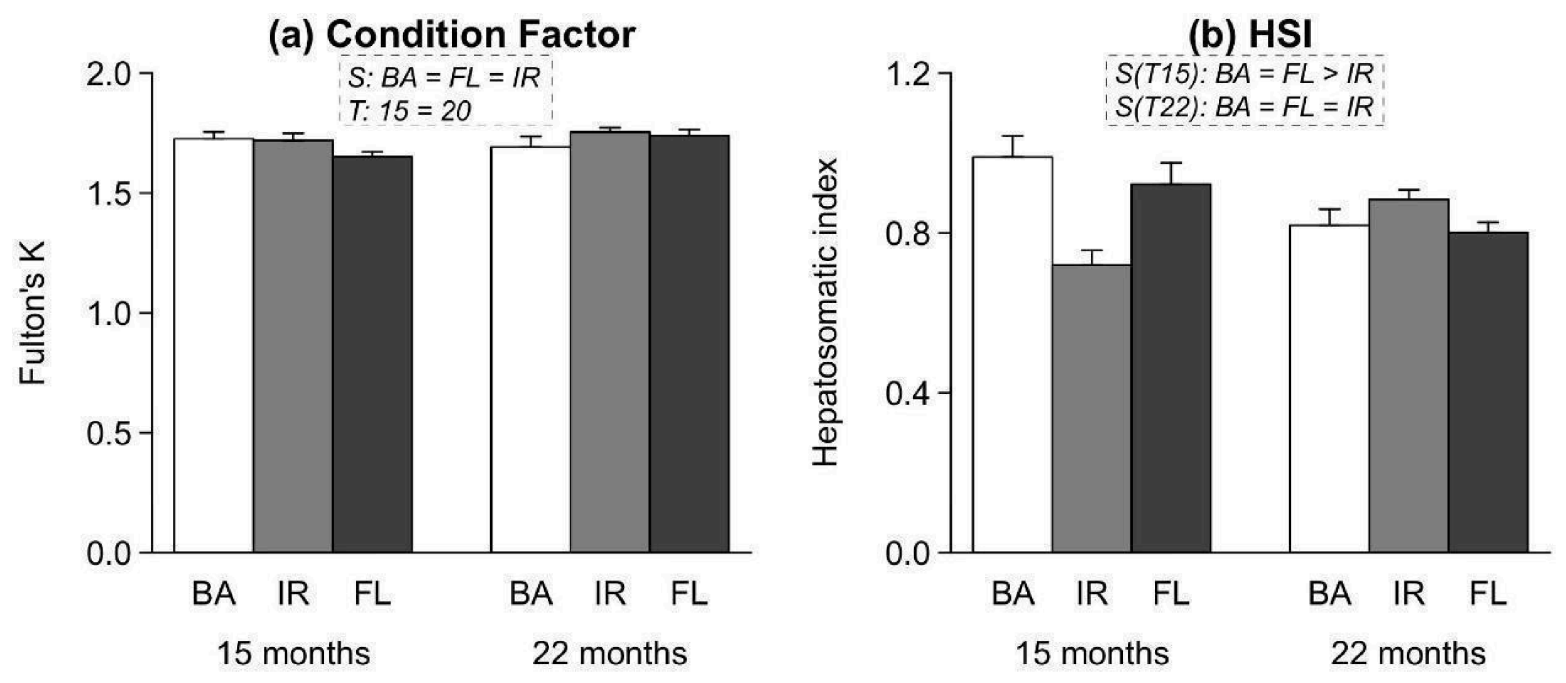
Somatic indices of *O. niloticus* (mean + SE). (**a**) Fulton’s condition factor (K) and (**b**) hepatosomatic index (HSI) of fish from the different exposure scenarios in the Segredo Reservoir after 15 and 22 months. BA = *Barragem*, IR = *Iratim*, FL = *Floresta*. Results from the SK post hoc tests are presented inside dotted boxes, indicating differences among the sampled exposure scenarios as larger (“>”), smaller (“<”) if *p* < 0.05 or equal (“=”) if *p* ≥ 0.05. The labels S(T15) and S(T22) indicate comparisons among the sampled exposure scenarios after 15 and 22 months, respectively.

The analysis of variance showed that the overall condition of the fish, assessed by Fulton’s K condition factor, did not differ among the sampled exposure scenarios (Figure 2a). In contrast, liver size varied among the in situ exposure scenarios. The hepatosomatic index (HSI) differed according to site and exposure time, with consistently higher values observed in fish from the *Barragem* and *Floresta* in situ exposure scenarios than in those from the *Iratim* exposure scenario. However, the differences observed among sites were significant only at 15 months of exposure and differences due to the interaction accounted for only 13.7% of HSI variance (Table S1); after 22 months, HSI values converged and no longer differed among the evaluated sites (Figure 2b). Full ANOVA results, together with Scott-Knott post hoc tests and effect size measures, are provided in the Supplementary Materials (Table S1).

### 3.2. Morphological Hepatotoxicity

**Figure 3:**
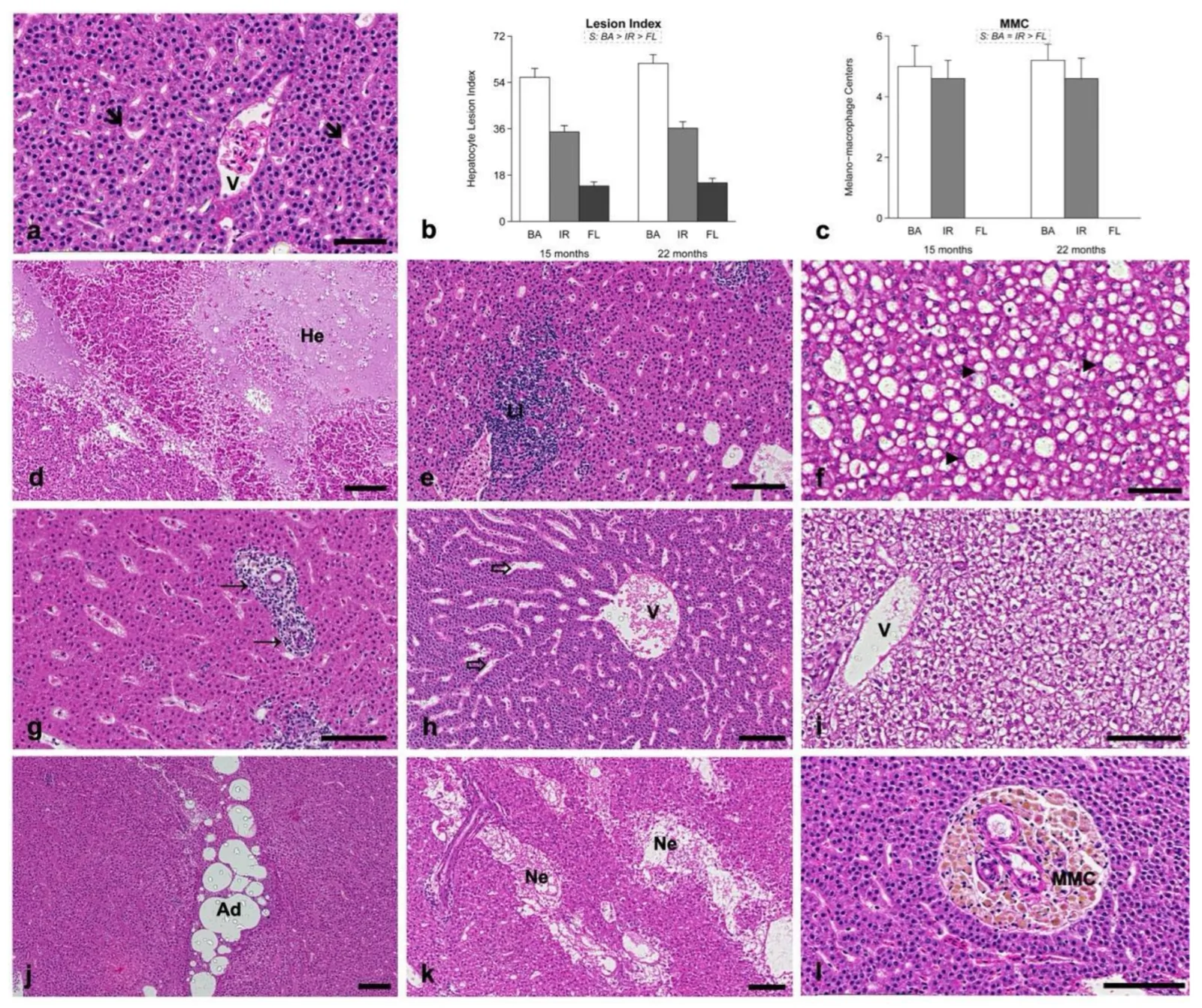
Histopathological alterations detected in the liver of *O. niloticus* (mean + SE). (**a**) Normal liver structure showing central vein (v) and sinusoids (➨). (**b**) Histopathological lesion index. (**c**) Melanomacrophage center index. Representative liver alterations in fish from different sampling sites: (**d**) *Barragem.* Hemorrhage area (He); (**e**) *Floresta*. Leukocyte infiltration (LI); (**f**) *Iratim*. Steatosis (►); (**g**) *Barragem.* Granulomatosis (→); (**h**) *Iratim.* Sinusoid dilation (**⇨**); (**i**) *Iratim*, Hepatocyte vacuolization (**j**) Iratim. Infiltration of adipocytes (Ad); (**k**) *Floresta.* Necrotic area (Ne); (**l**) *Barragem*. Melanomacrophage center (MMC). Hematoxylin and Eosin staining. Scale bars (50–100 μm). Results from the SK post hoc tests are presented inside dotted boxes, indicating differences among the sampled exposure scenarios as larger (“>”), smaller (“<”) if *p* < 0.05 or equal (“=”) if *p* ≥ 0.05. The labels S(T15) and S(T22) indicate comparisons among the sampled exposure scenarios after 15 and 22 months, respectively.

**Figure 4:**
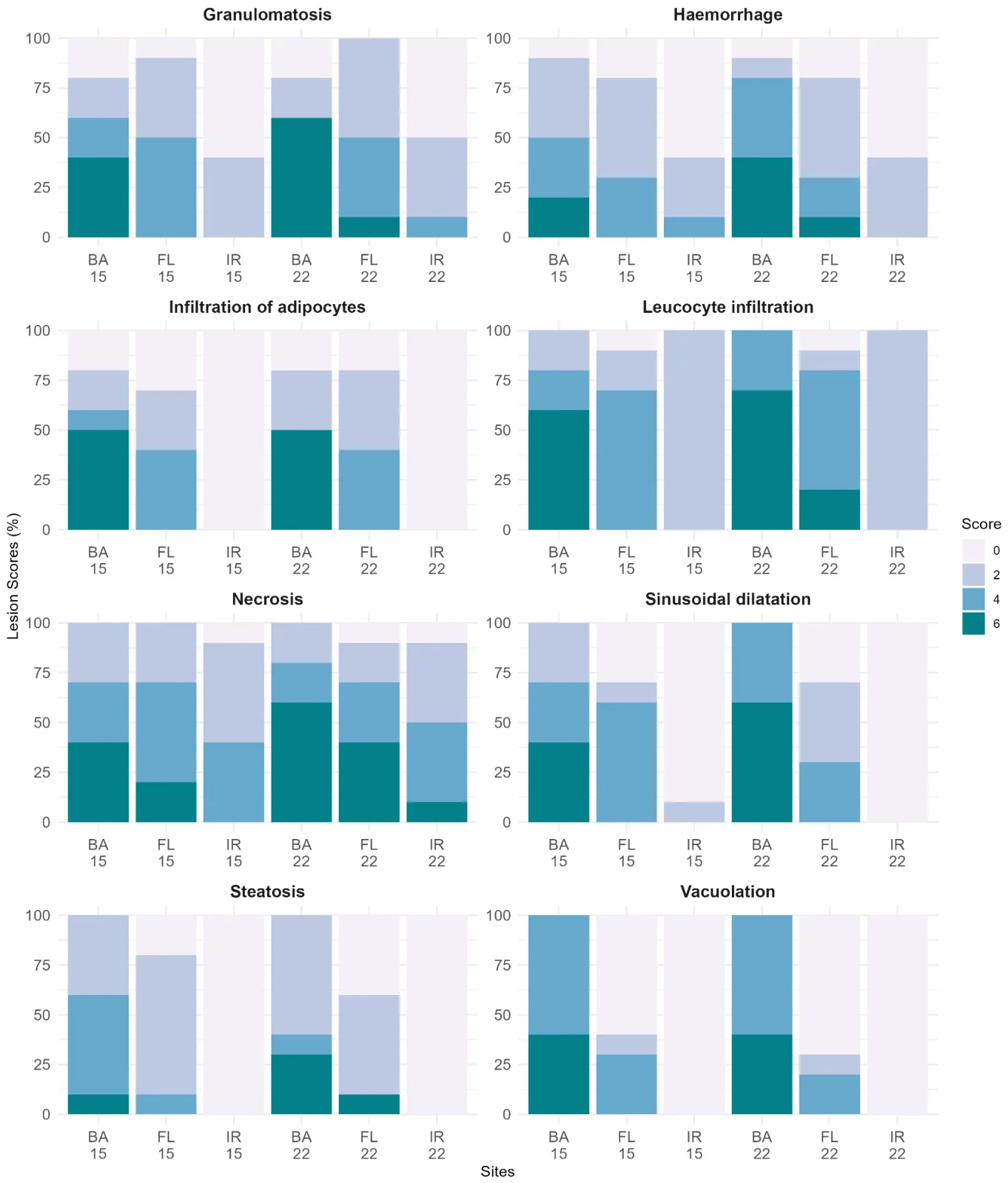
Distribution of histopathological lesion severity scores in fish maintained under the different in situ exposure scenarios in the Segredo Hydroelectric Reservoir, after 15 and 22 months. Histopathological alterations were classified into four severity categories: score 0 (absent), score 2 (mild), score 4 (moderate), and score 6 (severe). For each lesion and in situ exposure scenario, stacked bars represent the percentage of fish assigned to each severity category, with each bar totaling 100%. Higher contributions of scores 4 and 6 indicate a greater prevalence of moderate to severe lesions, whereas higher contributions of score 0 indicate a predominance of unaffected individuals. The sampled sites were *Barragem* (BA), *Floresta* (FL), and *Iratim* (IR).

The typical pattern of liver tissue, previously described for most teleost fish, consists of a homogeneous tissue with sinusoids and polyhedral hepatocytes, arranged in cords, with spherical nuclei, bile ducts, blood vessels and sinusoidal capillaries (Figure 3a). The liver lesion index was higher in fish from the *Barragem* in situ exposure scenario than in those from the *Iratim* and *Floresta* exposure scenarios (Figure 3b). In addition, MMCs were observed in fish from the *Barragem* and *Iratim* exposure scenarios (Figure 3c,l). All fish displayed notable histopathological changes, such as hemorrhage (Figure 3d), leukocyte infiltration (Figure 3e), steatosis (Figure 3f), peritubular granulomatosis (Figure 3g), sinusoidal dilatation (Figure 3h), vacuolation (Figure 3i), adipocyte infiltration (Figure 3j) and necrosis (Figure 3k). The scores assigned to these histopathological lesions are presented in Figure 4.

### 3.3. Liver Biochemical Analysis and Genotoxic Damage

**Figure 5:**
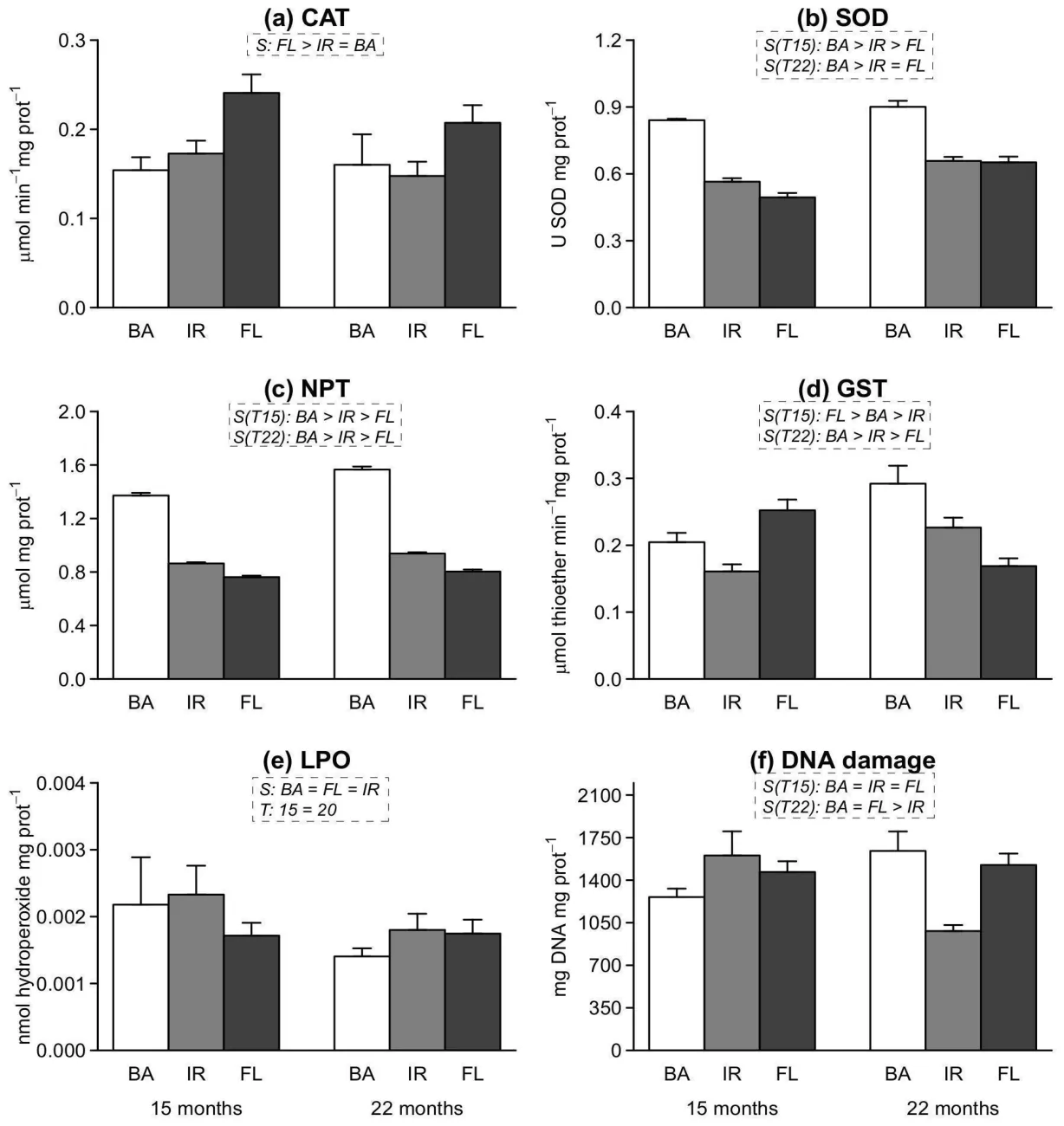
Biochemical biomarkers and DNA damage in the liver of *O. niloticus* (mean + SE). (**a**) Catalase (CAT) activity, (**b**) superoxide dismutase (SOD) activity, (**c**) non-protein thiol/glutathione (NPT) levels, (**d**) glutathione S-transferase (GST) activity, (**e**) lipid peroxidation (LPO), and (**f**) DNA damage in fish maintained under the different in situ exposure scenarios after 15 and 22 months (BA = *Barragem*, IR = *Iratim*, FL = *Floresta*). Results from the SK post hoc tests are presented inside dotted boxes, indicating differences among the sampled exposure scenarios as larger (“>”), smaller (“<”) if *p* < 0.05 or equal (“=”) if *p* ≥ 0.05. The labels S(T15) and S(T22) indicate comparisons among the sampled exposure scenarios after 15 and 22 months, respectively.

Most biochemical biomarkers showed differences among the sampled in situ exposure scenarios. CAT activity (Figure 5a) showed the highest values in fish from the *Floresta* exposure scenario compared with those from *Barragem* and *Iratim.* Conversely, SOD activity (Figure 5b), NPT levels (Figure 5c), GST activity (Figure 5d), and DNA damage (Figure 5f) were markedly higher at the *Barragem*, followed by *Floresta* and *Iratim* exposure scenarios. This pattern was consistent at both 15 and 22 months of exposure, except for SOD, which after 22 months remained highest at *Barragem*, whereas at *Floresta* and *Iratim* it stayed comparatively low. Despite the significant Site × Time interaction for SOD and NPT, effect size measures revealed that differences among sites accounted for the largest proportion of total variance, explaining 59% and 92% of differences in SOD activity and NPT levels, respectively. Finally, LPO levels (Figure 5e) did not differ significantly among the sampled exposure scenarios. Full ANOVA results, Scott–Knott post hoc tests and effect size measures are provided in the Supplementary Materials (Table S1).

### 3.4. Plasma Biochemical Parameters

**Figure 6:**
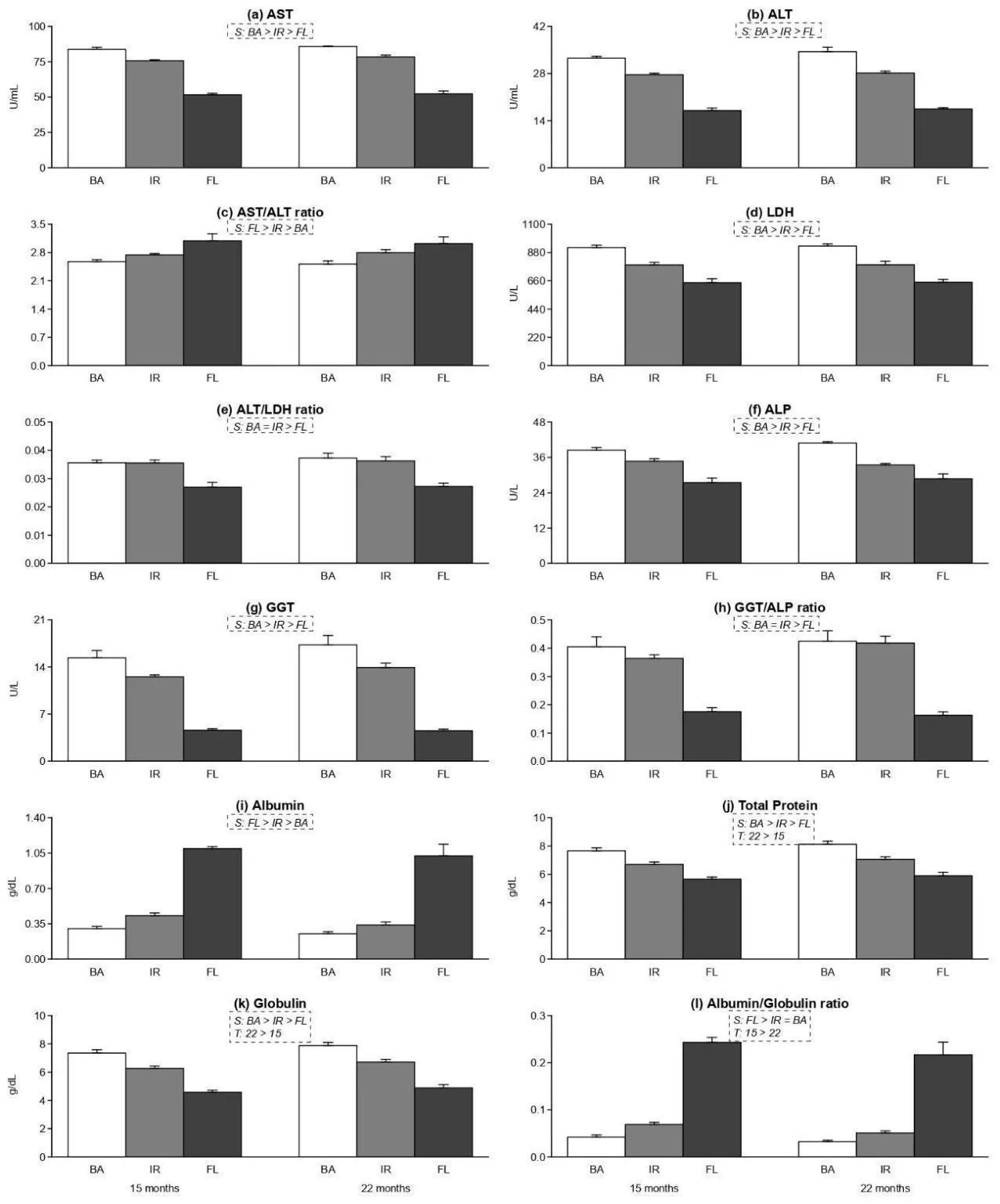
Plasma biochemical parameters of *O. niloticus* (mean + SE). (**a**) aspartate transaminase (AST), (**b**) alanine transaminase (ALT), (**c**) AST/ALT ratio, (**d**) lactate dehydrogenase (LDH), (**e**) ALT/LDH ratio, (**f**) alkaline phosphatase (ALP), (**g**) gamma glutamyltransferase (GGT), (**h**) GGT/ALP ratio, (**i**) albumin, and (**j**) total protein, (**k**) globulin, and (**l**) albumin/globulin ratio after 15 and 22 months in BA = *Barragem*, IR = *Iratim*, FL = *Floresta*. Results from the SK post hoc tests are presented inside dotted boxes, indicating differences among the sampled exposure scenarios as larger (“>”), smaller (“<”) if *p* < 0.05 or equal (“=”) if *p* ≥ 0.05. The labels S(T15) and S(T22) indicate comparisons among the sampled exposure scenarios after 15 and 22 months, respectively.

Plasma biochemical assays enabled the evaluation of different parameters related to tissue integrity and metabolism. Most plasma parameters were higher in fish from the *Barragem* in situ exposure scenario, followed by those from the *Iratim* and *Floresta* exposure scenarios, as shown in Figure 6a,b,d,f,g. This pattern was observed at both exposure durations (15 and 22 months). Conversely, the AST/ALT ratio (Figure 6c) and albumin levels (Figure 6i) were higher in fish from the *Floresta* exposure scenario, whereas the ALT/LDH ratio (Figure 6e) and the GGT/ALP ratio (Figure 6h) were similarly elevated in fish from the *Barragem* and *Iratim* exposure scenarios compared with those from the *Floresta* exposure scenario. Total protein levels (Figure 6j) showed differences among the sampled exposure scenarios, with the highest values observed in fish from the *Barragem* exposure scenario, followed by those from the *Iratim* and *Floresta* exposure scenarios. Overall, protein levels were higher after 22 months of exposure. Similarly, globulin levels (Figure 6k) were highest in fish from the *Barragem* exposure scenario, followed by those from the *Iratim* and *Floresta* exposure scenarios, after 22 months of exposure. Finally, the albumin/globulin ratio (Figure 6l) was higher in fish from the *Floresta* exposure scenario than in those from the *Iratim* and *Barragem* exposure scenarios after 15 months of exposure. Overall, differences among sites accounted for the largest proportion of total variance in plasma biochemical parameters, contributing more than 62% to the variability in 10 out of 12 parameters analyzed. Detailed results of the ANOVA, post hoc tests and effect size measures are provided in the Supplementary Materials (Table S2).

### 3.5. Chemical Analysis of Water, Sediment, and Tissue

In the water samples, only metals exceeded the limits established by Brazilian legislation for freshwater bodies, including the Iguaçu River. Specifically, elevated concentrations of aluminum (Al) were observed at *Floresta* and *Iratim* exposure scenarios, cadmium (Cd) at *Barragem* and *Floresta* exposure scenarios, iron (Fe) only at *Floresta* exposure scenario, and arsenic (As) in all exposure scenarios (Supplementary Materials, Table S4). Overall, most of the remaining contaminants analyzed in this study were detected either below the limit of detection or within the regulatory limits established by Brazilian legislation [50]. Sediment samples contained polycyclic aromatic hydrocarbons (PAHs), including phenanthrene, fluoranthene, pyrene, benz[a]anthracene, and C1-naphthalene, all of which exceeded the established limits at *Floresta* exposure scenario (Supplementary Materials, Table S3). Chemical analysis of fish muscle revealed the presence of PAHs, such as naphthalene and pyrene, at all sampling sites during both exposure periods. The *Floresta* exposure scenario showed the highest levels after 15 months, whereas the *Iratim* exposure scenario exhibited the highest levels after 22 months. Additionally, polychlorinated biphenyls (PCBs) were detected in all exposure scenarios during both exposure periods. Since there are no specific regulations establishing limits for pollutants in fish tissues, these results can only be compared among the sampled exposure scenarios.

### 3.6. Principal Component Analysis: Integrated Perspective of Biomarker Variation

**Figure 7:**
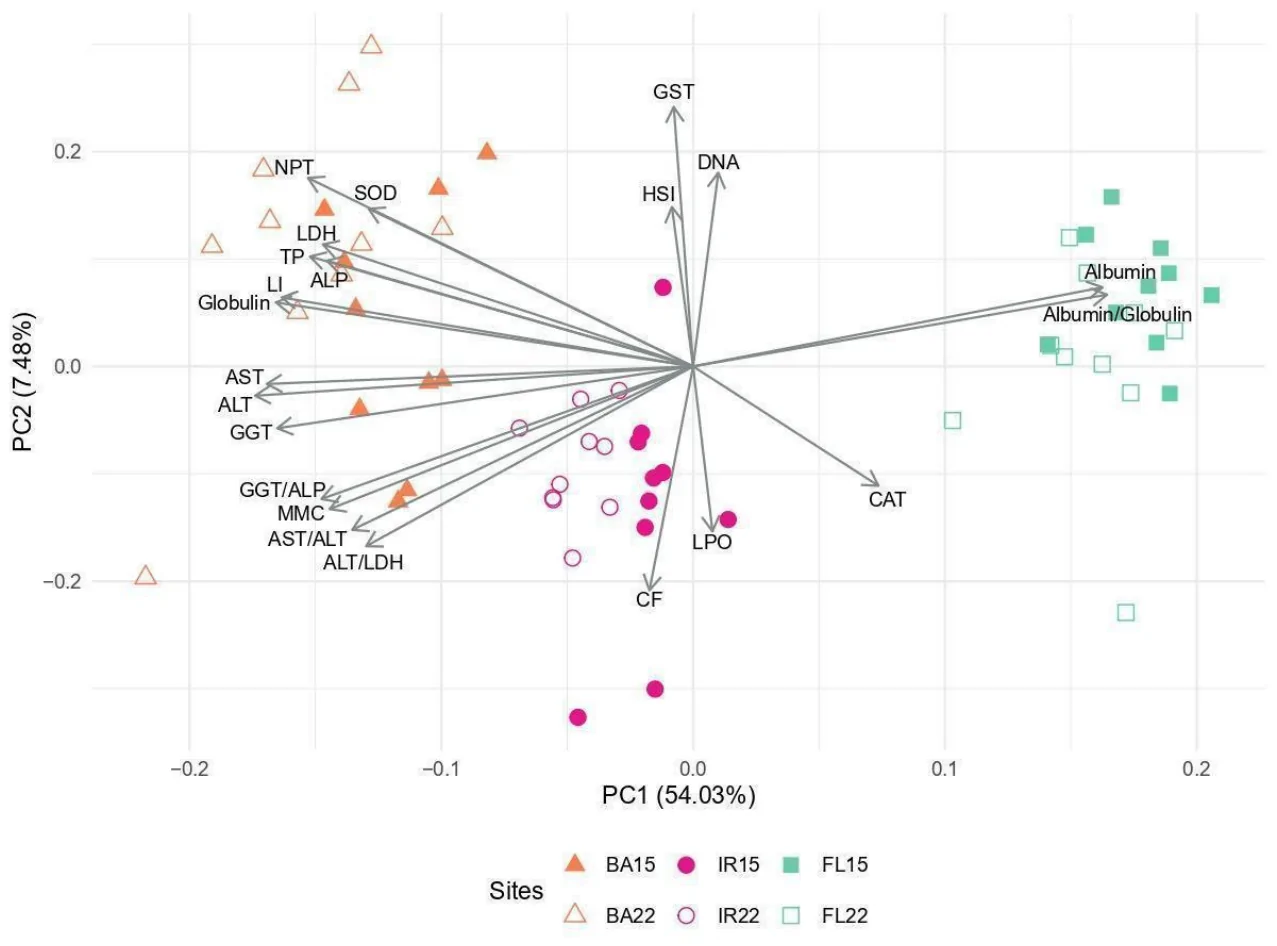
Principal component analysis (PCA) showing the distribution of fish across the sampled exposure scenarios in the Segredo Hydroelectric Reservoir and their association with the measured biochemical biomarkers. *Barragem* at 15 months (BA 15, filled triangles), *Barragem* at 22 months (BA 22, open triangles), *Floresta* at 15 months (FL 15, filled circles), *Floresta* at 22 months (FL 22, open circles), *Iratim* at 15 months (IR 15, filled squares) and *Iratim* at 22 months (IR 22, open squares). Biochemical biomarkers: catalase (CAT), superoxide dismutase (SOD), non-protein thiol/glutathione (NPT), glutathione *S*-transferase (GST), lipid peroxidation (LPO), DNA damage (DNA), aspartate transaminase (AST), Alanine transaminase (ALT), AST/ALT ratio, lactate dehydrogenase (LDH), Alkaline phosphatase (ALP), Gamaglutamiltransferase (GGT), GGT/ALP ratio, albumin, total protein (TP), globulin, albumin/globulin ratio, histopathological lesion index (LI), hepatosomatic index (HSI), and Fulton’s condition factor (CF).

The principal component analysis (PCA) explained 61.5% of the total variance, of which 54.0% corresponded to PC1 and 7.5% to PC2 (Figure 7). The PCA revealed distinct multivariate patterns among the sampled exposure scenarios based on the measured biomarker responses. The *Barragem* exposure scenario was characterized by elevated levels of several plasma parameters and a higher histopathological lesion index, as indicated by the clustering of variables such as LDH, TP, ALP, AST, ALT, and GGT. In contrast, the *Floresta* exposure scenario was characterized by the positioning of albumin and the albumin/globulin ratio. The *Iratim* site was predominantly associated with biomarkers such as CAT, GST, HSI, DNA damage, condition factor, and LPO. However, the proximity of these variables to the plot origin indicates relatively weaker contributions to the first two principal components and should not be interpreted as evidence of specific associations with a particular exposure scenario or exposure duration.

### 3.7. Hepatic Responses Under Chronic Exposure to the Iguaçu River

**Figure 8:**
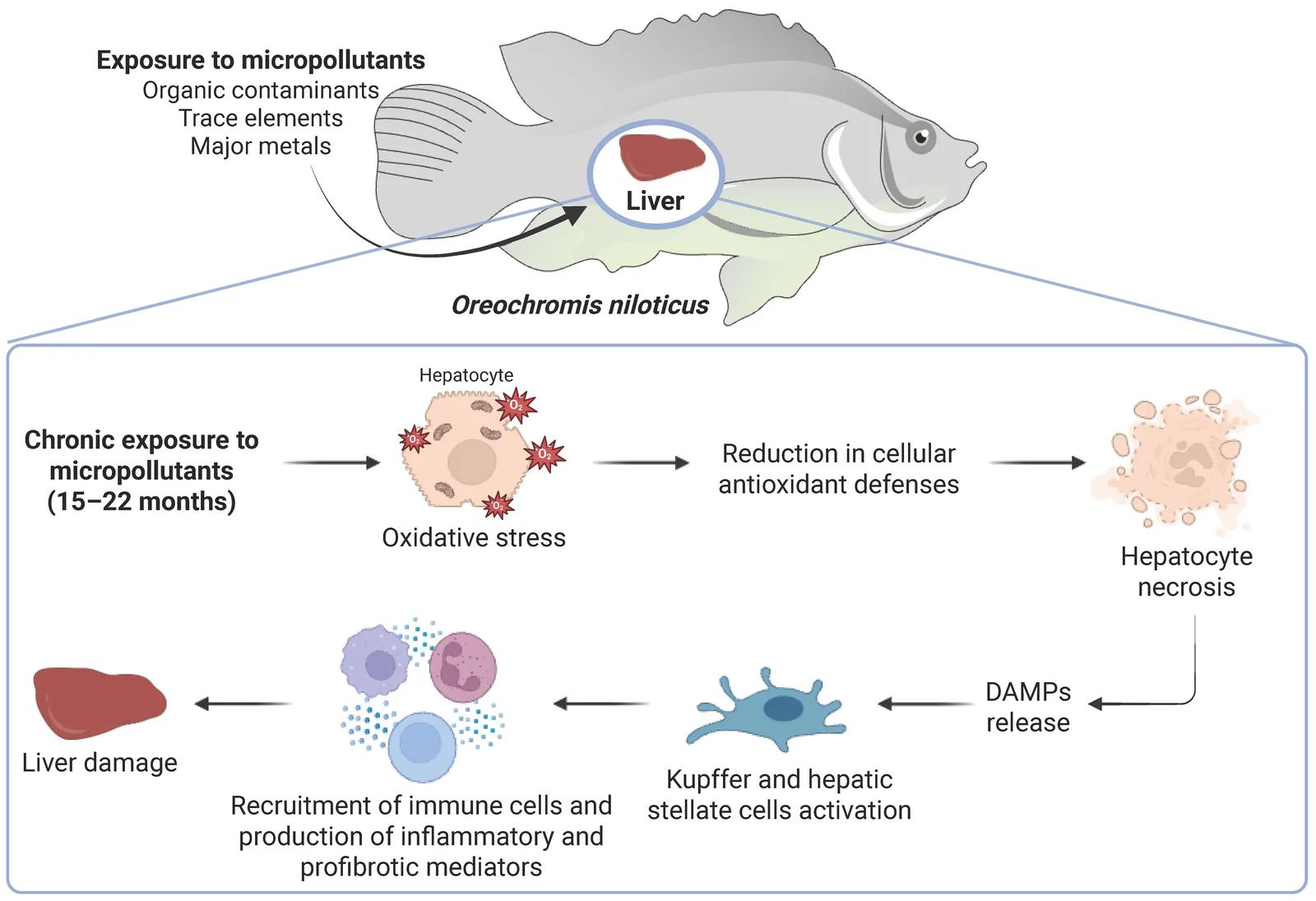
Conceptual model illustrating the proposed biological pathways associated with the hepatic responses observed in *O. niloticus* under the evaluated exposure scenarios in the Iguaçu River. Created in BioRender.com.

Based on the integrated analysis of all evaluated variables, a hypothetical sequence of biological responses associated with the monitored environmental exposure scenarios is proposed in Figure 8, integrating findings from the present study with mechanisms described in the scientific literature. The liver is a crucial organ for the detoxification and elimination of drugs and toxins from the body. The oxidative stress observed in the present study is consistent with chronic exposure to complex environmental contaminant mixtures in the Iguaçu River. Exposure may increase the production of reactive oxygen and nitrogen species (ROS and RNS), potentially overwhelm cellular antioxidant defenses, and contribute to the disruption of redox homeostasis. This oxidative stress aligns with mechanisms described for chronic contaminant exposure, in which excessive ROS and RNS damage cellular macromolecules, contributing to hepatocyte injury and necrosis [51]. Based on mechanisms proposed in the literature, hepatocyte injury may be accompanied by the release of damage-associated molecular patterns (DAMPs), which can activate resident liver cells, including Kupffer and hepatic stellate cells, and potentially amplify local inflammatory responses. Previous studies have shown that these processes may promote immune cell recruitment and the release of inflammatory and profibrotic mediators, potentially contributing to the progression of liver injury [52]. However, DAMP release, specific resident-cell activation, immune cell recruitment, and the release of inflammatory or profibrotic mediators were not directly measured in the present study and are therefore presented as hypothetical intermediate pathways in Figure 8. In the present study, most evaluated biomarkers showed significant differences among the sampled exposure scenarios, with several responses being more pronounced after 22 months than after 15 months. These patterns are consistent with an increase in the magnitude of biological responses over the exposure period; however, given the lack of cage-level replication, they should be interpreted as exploratory patterns rather than independently replicated temporal effects.

## 4. Discussion

In fish, the liver plays a central role in detoxification, biotransformation, and metabolism, making it a sensitive organ for assessing environmental contamination [17,18,27,31,42]. In the present study, hepatic biomarker responses differed among the evaluated exposure scenarios, aligning with structural and functional alterations associated with distinct water quality profiles. Nile tilapia maintained at the Iguaçu River monitoring sites exhibited marked alterations in liver structure and function, with the most pronounced responses observed in fish from the *Barragem* scenario at both 15 and 22 months. These findings suggest that long-term exposure to complex mixtures of environmental contaminants may be associated with multilevel biological responses, ranging from biochemical and cellular alterations to broader physiological effects, consistent with the biological consequences of environmental degradation of the reservoir.

The absence of significant differences in Fulton’s condition factor among the sampled in situ exposure scenarios indicates that no marked differences in overall body condition were detected among the monitored groups, despite evidence of hepatic alterations. However, fish from the *Barragem* and *Floresta* exposure scenarios showed higher HSI values after 15 months of exposure, a pattern that may be consistent with enhanced hepatic metabolic and biotransformation activity under chronic exposure to environmental contaminants. According to previous studies [8,53], changes in the HSI are frequently related to the presence of organic pollutants. Similarly, it has been reported that liver enlargement (via hypertrophy and hyperplasia) can occur in individuals from contaminated sites as a response to the increased metabolic demand for detoxification [53,54]. Interestingly, these differences were not observed after 22 months of exposure. This pattern may reflect physiological compensation or adaptation during prolonged environmental exposure. However, these mechanisms were not directly assessed in the present study. Chronic exposure can promote compensatory metabolic adjustments and changes in energy allocation that help maintain physiological homeostasis over time, while HSI may also be influenced by seasonal and nutritional factors [15,53], which could contribute to this temporal variability. Therefore, the apparent convergence of HSI values after 22 months should not be interpreted as evidence of recovery. Rather, it highlights a metabolic compensation that occurs while histopathological lesions, oxidative stress responses, and DNA damage remain evident, indicating that altered hepatic function persisted despite the stabilization of this somatic index.

The interpretation of these somatic indexes can be complemented when combined with histopathological analysis, allowing for more comprehensive and insightful comparisons among sites. Alterations in normal tissue structure provide valuable information about the integrated effects of molecular, biochemical, and physiological changes associated with environmental exposure to complex contaminant mixtures [55]. Despite possible compensatory or adaptive responses, severe histopathological lesions, including cytoplasmic vacuolation, necrosis, steatosis, sinusoidal dilatation, hemorrhage, and inflammatory infiltration, were observed across the monitored exposure scenarios, with more pronounced alterations observed in fish from the *Barragem* exposure scenario. Hepatocyte vacuolation and steatosis indicate metabolic disturbances related to lipid accumulation and disrupted energy regulation [56,57,58].

Hepatocyte vacuolation is frequently reported as a histopathological alteration in fish exposed to environmental contaminants and has been associated with exposure to compounds such as bisphenol A, organochlorines, PCBs, petroleum hydrocarbons, estrogens, and cyanobacterial toxins [59,60]. Previous studies have suggested that lipid accumulation may result from reduced cellular enzymatic activity, impairing lipid metabolism [12]. Cases of steatosis are common in fish exposed to environmental pollution [3,61]. In this study, the occurrence of necrotic areas and sinusoidal dilatation in fish maintained under the exposure scenarios is consistent with hepatic alterations reported in association with chronic environmental exposure to complex contaminant mixtures. These alterations have been associated with oxidative membrane damage and vascular stasis in previous studies [62,63]. However, these mechanisms were not directly assessed in the present study, and the observed histopathological alterations do not allow contaminant-specific mechanistic conclusions. Similar histopathological findings have been reported in fish from the Iguaçu River exposed to environmental pollutants [3,4,17,28].

Biochemical biomarkers indicated the occurrence of oxidative stress and detoxification responses that were consistent with the histological alterations observed [64,65]. The elevated activities of SOD, GST, and CAT, together with increased NPT levels, are consistent with the activation of antioxidant defense mechanisms commonly described in response to oxidative stress. Similar responses have been reported in fish exposed to complex mixtures of environmental contaminants, including PAHs, PCBs, and trace metals [66,67]. Differences among the in situ exposure scenarios were also observed, with higher SOD and GST activities in fish from the *Barragem* and higher CAT activity in fish from the *Floresta* exposure scenario. These patterns may reflect differences in environmental conditions, including contaminant mixtures and their bioavailability, among the sampled exposure scenarios [68,69,70,71]. Nevertheless, the persistence of oxidative stress may have exceeded the capacity of the antioxidant defense system, as reflected by the increased DNA damage observed at *Barragem* after 22 months of exposure. This finding is consistent with alterations in cellular and genomic integrity under prolonged environmental exposure. However, the present study does not establish a direct mechanistic link between oxidative stress and DNA damage. Likewise, the biochemical and genotoxic responses occurred alongside histopathological alterations, including cytoplasmic degeneration and necrosis, which may be biologically related to oxidative imbalance, although this relationship was not directly demonstrated in the present study.

Inflammatory responses were also prominent, as shown by leukocyte infiltration, the presence of MMCs, and granuloma formation. These alterations are consistent with persistent immune activation commonly associated with chronic tissue injury and environmental contaminant exposure. The increased occurrence of MMCs, especially at the *Barragem* and *Iratim* exposure scenarios, may represent an adaptive or compensatory response involved in the sequestration and degradation of damaged cells and foreign material, as previously described for fish exposed to contaminated environments [72,73]. Extensive research demonstrates a link between the proliferation of melanomacrophages and exposure to various pollutants, including metals [10,72,73,74,75], antifouling agents [42] and persistent organic pollutants [76,77]. Similarly, the occurrence of granulomas is consistent with chronic inflammatory responses reported in fish exposed to persistent or non-degradable agents, which may involve macrophage activation and the release of inflammatory mediators [78,79,80,81]. The combined occurrence of necrosis, melanomacrophages, and granulomas in fish from the sampled exposure scenarios is consistent with sustained inflammatory responses under chronic environmental exposure to complex contaminant mixtures, including metals and persistent organic pollutants. However, specific immune activation pathways, including macrophage activation and cytokine release, were not directly assessed in the present study. Overall, the extensive inflammatory changes observed in the liver are consistent with ongoing tissue injury, although the contribution of individual contaminants cannot be distinguished under the conditions of the present study [57].

Plasma biochemical results were consistent with altered hepatic function. Increased AST, ALT, and LDH activities, particularly in fish from the *Barragem* exposure scenario, are commonly associated with hepatocellular injury and metabolic stress [82,83]. Although these values remained within the physiological range reported for the species [40], the higher AST/ALT ratio (>2.0) has been associated in previous studies with hepatic alterations, including fibrosis [84]; however, this condition was not directly assessed in the present study. Similarly, the higher LDH activity is consistent with tissue injury and may be associated with altered cellular metabolism or tissue stress, in conjunction with the histopathological lesions observed. Changes in protein metabolism were also evident. Previous studies have shown that reduced albumin levels accompanied by increased globulin and total protein concentrations are commonly associated with inflammatory and immune responses [85,86]. A similar pattern was observed in the present study, which may reflect alterations in hepatic protein metabolism associated with chronic environmental exposure. However, the mechanisms underlying these changes, including potential compensatory responses, were not directly assessed in the present study.

The integration of biochemical, histopathological, and physiological biomarkers provides a comprehensive view of the biological responses associated with chronic environmental exposure in fish. The alignment among antioxidant enzyme activities, histopathological alterations, and plasma biochemical changes is consistent with oxidative stress responses, inflammatory alterations, and changes in hepatic function under the evaluated environmental exposure scenarios. Together, these biomarkers provide complementary evidence of the biological effects of chronic environmental contamination. Despite the apparent maintenance of somatic indices, these findings do not necessarily indicate the absence of biological effects. Previous studies suggest that, under chronic environmental exposure, fish may maintain their overall condition by reallocating energy resources to detoxification and tissue repair, potentially at the expense of growth and reproduction [87]. However, such energy-allocation mechanisms were not directly assessed in the present study. From an ecological perspective, these responses may have implications for organismal performance and fitness if sustained over prolonged periods, although such consequences were not directly evaluated here. Chronic exposure to a mixture of metals (Cd, Pb, Al), metalloids (As), and organic contaminants, including PAHs and PCBs, previously reported [4] at the same sampling locations, provides an environmental context consistent with the biological responses observed in the present study. Although the present study did not assess reproductive success, survival, or population dynamics, the combination of oxidative stress responses, genotoxic damage, and histopathological alterations can be considered a relevant early warning signal of potential adverse biological consequences. Such sublethal alterations may affect organismal performance and, if sustained, could potentially contribute to broader ecological consequences; however, population-level effects cannot be inferred from the present study. These findings highlight the importance of integrating responses across biological levels to understand pollutant impacts not only at the organismal level but also within broader ecological contexts.

Although the present study was not designed to establish direct cause-and-effect relationships between individual contaminants and specific biological responses, the integration of chemical and biological data provides important insights into the biological responses observed under the exposure scenarios and their relationship with chronic exposure to complex environmental mixtures. Previous surveys conducted at the same sampling sites reported the occurrence of metals, metalloids, PAHs, and PCBs [4], and similar contaminant classes were detected in water, sediment, and fish tissues in the present study. However, the contaminants analyzed in the present study do not represent the full spectrum of environmental pollutants potentially present in these aquatic systems. Most measured compounds were below regulatory thresholds, and sampling sites with the highest concentrations of individual contaminant classes were not always those exhibiting the strongest biological responses. Therefore, the observed hepatic alterations should be interpreted as integrated responses associated with chronic exposure to complex contaminant mixtures, including contaminants not evaluated in the present study, contaminant bioavailability, and other environmental stressors inherent to field conditions, rather than as effects attributable to specific pollutants. The pronounced hepatic damage, oxidative stress, immune activation, and genotoxic responses observed at the *Barragem* exposure scenario after 22 months are consistent with sustained biological responses under prolonged environmental exposure. However, the present study does not allow for determining whether these responses resulted from the exhaustion of compensatory mechanisms or from cumulative cellular injury. Likewise, differences in antioxidant responses among sites may reflect physiological adjustments to distinct environmental exposure scenarios rather than responses to a single contaminant class. Overall, these findings reinforce that biomarker responses in active biomonitoring studies represent the combined effects of multiple interacting factors, and the conceptual model presented in Figure 8 should be interpreted as a hypothetical framework of biological interactions under complex environmental exposure conditions rather than evidence of direct causality.

Although this study provides ecologically relevant evidence of chronic biological stress in fish maintained under the evaluated exposure scenarios in the Iguaçu River, some limitations should be considered when interpreting the findings. The absence of replicated cages within each site × exposure-time combination precludes fully replicated statistical inference at the experimental-unit level. Consequently, the observed differences should be interpreted as biomarker responses among individual fish under specific exposure scenarios rather than as evidence of manipulative treatment effects. Nevertheless, because the net cages functioned solely as standardized containment structures while allowing continuous river water circulation, the study reflects environmentally realistic exposure conditions and provides biologically relevant information for active biomonitoring under natural field conditions. In addition, because continuous environmental monitoring data (e.g., temperature, rainfall, dissolved oxygen, and food availability) were not available throughout the entire exposure period, the potential influence of seasonal and environmental variation on biomarker responses cannot be completely excluded. Another limitation of this study is the absence of a truly uncontaminated reference site within the Segredo Reservoir. Because all sampling sites are subject to different degrees of anthropogenic influence, the present study was designed to compare biological responses among distinct environmental exposure scenarios rather than against pristine baseline conditions. Consequently, the observed differences should be interpreted as relative patterns among exposure scenarios and not as absolute deviations from uncontaminated reference values. Moreover, reproductive and endocrine endpoints were not assessed, which could further elucidate long-term ecological consequences and should be evaluated in future studies. Therefore, the biological alterations reported here should be interpreted as indicators of environmental exposure and chronic stress across the evaluated exposure scenarios, rather than as evidence of direct causality attributable to specific pollutants. Collectively, these multilevel biological responses provide complementary evidence of chronic environmental stress across the evaluated exposure scenarios, while their interpretation remains conditional on the study design and the absence of cage–level replication.

## 5. Conclusions

In summary, the liver of caged *O. niloticus* exhibited biomarker responses consistent with chronic environmental exposure under the different exposure scenarios evaluated in the Iguaçu River. Overall, the observed biomarker responses were associated with the environmental exposure scenarios evaluated rather than with specific contaminant classes, reflecting the complexity of chronic exposure under field conditions. The integration of histopathological, biochemical, and physiological biomarkers indicates that oxidative stress, inflammation, and metabolic changes occurred alongside alterations in hepatic structure and function under the evaluated exposure scenarios. Although these hepatic alterations provide evidence of biological responses associated with environmental stressors, chemical concentration data alone do not fully capture the biological impacts on aquatic organisms, highlighting the value of integrating multiple biomarkers for comprehensive environmental assessment. The observed patterns across the sampled exposure scenarios and exposure durations further demonstrate the value of evaluating multiple biological levels when characterizing responses under chronic environmental exposure. Overall, these findings support the use of an integrated multibiomarker approach to assess the biological consequences of long-term environmental exposure to complex contaminant mixtures. Continued environmental monitoring, improvements in wastewater treatment, and further investigation of contaminant mixtures and their interactions are essential to support ecological risk assessment and the conservation of the Iguaçu River Basin.

## Data Availability

The original contributions presented in this study are included in the article/Supplementary Materials. Further inquiries can be directed to the corresponding authors.

## Supplementary Materials

The following supporting information can be downloaded at: https://www.mdpi.com/article/10.3390/jox16050162/s1. Supplementary Methods S1. Detailed histological procedures and lesion scoring criteria; Table S1: Analyses of variance of hepatic biochemical biomarkers of fish sampled at three distinct Sites (S): Barragem (BA), Floresta (FL) and Iratim (IR) after 15 months of exposure (Time, T15) and 22 months (Time, T22); Table S2: Analyses of variance of biochemical biomarkers assessed from plasma of fish sampled at three distinct Sites (S): Barragem (BA), Floresta (FL) and Iratim (IR) after 15 months of exposure (Time, T15) and 22 months (Time, T22); Table S3: Organic pollutants detect in water, sediment and muscle of fish sampled at Barragem (BA), Floresta (FL) and Iratim (IR) sites. Muscle samples were obtained at 15 months and 22 months of exposure to reservoir waters; Table S4: Trace elements detect in water and sediment sampled at Barragem (BA), Floresta (FL) and Iratim (IR) sites; File S1: Original images of Figure 3.
