# Supplementary material for: Monitoring Hepatic Biomarker Responses in Caged Oreochromis niloticus Under Chronic Exposure to Micropollutants in the Iguaçu River
Source: J Xenobiot. 2026 Aug 27;16(5):162. doi: 10.3390/jox16050162 (PMC13608329; doi:10.3390/jox16050162)
Supplement: Supplementary file 1 [file jox-16-00162-s001.zip › jox-4443389-supplementary.pdf]

Supplementary Materials: Monitoring Hepatic Biomarker Responses in Caged *Oreochromis niloticus* Under Chronic Exposure to Micropollutants in the Iguaçu River

Lorena Bavia, Rayanne Seibel Littig, Manuela Santos Santana, Milena Carvalho Carneiro, Luiza Santos Barreto, Thaís Muniz Vasconcelos, Marco Antonio Ferreira Randi, Cesar Castro Martins, Andrea Pinto De Oliveira, Iracema Opuskevitch, Fernando Cesar Alves Da Silva Ferreira, Juan Esquivel-Muelbert, Ciro Alberto De Oliveira Ribeiro and Maritana Mela Prodocimo

Supplementary Methods S1 – Histopathological procedures

Histopathological evaluation was performed using a semiquantitative scoring system based on the method described by [42], in which alterations were classified according to lesion severity and determined based on lesion severity. Histopathological alterations were scored from 0 to 3, where 0 = no alteration, 1 = slight alteration, 2 = moderate alteration and 3 = severe alteration. Slight alteration implies changes that do not cause damage to the hepatic tissue, considering that recovery of the liver can occur with the restoration of normal environmental conditions. Moderate alteration implies changes that are more severe and produce negative effects that could affect the functioning of the organ. Although lesions are repairable, if damaged areas increase or if tissues are chronically exposed to pollution, they may progress to severe alterations. In the severe alterations the restoration of the architecture of the liver is not possible so that the observed damage is irreversible. Each damage was scored from 0 to 6, reflecting the degree and extent of alteration (lesion) with 0 = < 10%, 2 = 10 - 40%, 4 = >40 - 70% and 6 = >70 - 100%. Pathological importance factor, assessing how the lesion impacted organ function and influenced fish survival was scored from 1 = easily reversible and of minimal pathological importance to 3 = generally irreversible and of marked importance. The Lesion Index was calculated as $\sum rp \sum alt\ (a \times w)$, where rp = reaction pattern, alt = alteration, a = lesion extension, and w = importance factor. Melanomacrophages centers (MMC) were counted by a checkerboard

test system coupled to one of the microscope eyepieces, whose area was 1 mm^2 where each square measured 0.01 mm^2. Fifteen randomly chosen fields were analyzed on slides of anterior liver of all individuals used in the study, stained with hematoxylin and eosin through light microscopy. The number of MMC was considered as those that contained 5 or more pigmented cells. The results of the melanomacrophage count were presented in mm^2 [43].

Supplementary Tables

Table S1. Analyses of variance of hepatic biochemical biomarkers of fish sampled at three distinct Sites (S): Barragem (BA), Floresta (FL) and Iratim (IR) after 15 months of exposure (Time, T15) and 22 months (Time, T22). Effect sizes measures (ω^2) are presented for each term of the ANOVA model. For Scott-Knott pairwise *a posteriori* comparisons: "<" and ">" indicate $P < 0.05$ and "=" indicates $P > 0.05$. Significant terms used in pairwise tests ($\alpha = 0.05$) and those that contributed to the largest proportion of total variance are highlighted in bold.

	**df**	**MS**	***F***	***P***	**ω^2**
(a) Catalase					
Site (S)	2	0.050	7.265	**0.001**	**0.106**
Time (T)	1	0.009	1.298	0.257	0.003
S × T	2	0.004	0.531	0.590	0.000
Residuals	100	0.007			
Scott-Knott tests	**S:** FL > IR = BA				
(b) Superoxide dismutase (log)					
Site (S)	2	0.358	119.728	<0.001	**0.591**
Time (T)	1	0.116	38.937	<0.001	0.094
S × T	2	0.012	4.108	**0.019**	0.015
Residuals	114	0.003			
Scott-Knott tests	**S(T15):** BA > IR > FL				
	**S(T22):** BA > IR = FL				
(c) Non-protein thiols (log)					

Site (S)	2	1.183	1193.09	<0.001	**0.923**
Time (T)	1	0.066	66.42	<0.001	0.025
S × T	2	0.008	8.26	**<0.001**	0.006
Residuals	114				
Scott-Knott tests	**S(T15):** BA > IR > FL				
	**S(T22):** BA > IR > FL				
(d) Glutathione S-transferase (√)					
Site (S)	2	0.022	4.494	0.013	0.044
Time (T)	1	0.011	2.141	0.146	0.007
S × T	2	0.094	18.998	**<0.001**	**0.228**
Residuals	108				
Scott-Knott tests	**S(T15):** FL > BA > IR				
	**S(T22):** BA > IR > FL				
(e) Lipid Peroxidation					
Site (S)	2	0	0.394	0.675	0.000
Time (T)	1	0	1.620	0.206	0.055
S × T	2	0	0.553	0.553	0.000
Residuals	109	0			
Scott-Knott tests	**S:** BA = FL = IR				
	**T:** 15 = 22				
(f) DNA damage (log)					
Site (S)	2	0.496	4.825	0.010	0.055
Time (T)	1	0.085	0.827	0.365	0.000
S × T	2	0.960	9.346	**0.002**	**0.120**
Residuals	109				
Scott-Knott tests	**S(T15):** BA = FL = IR				
	**S(T22):** BA= FL > IR				
(g) Hepatosomatic index (log)					

Site (S)	2	0.034	3.868	0.024	0.041
Time (T)	1	0.006	0.629	0.430	0.000
S × T	2	0.091	10.545	**<0.001**	**0.137**
Residuals	109				
Scott-Knott tests	**S(T15):** BA = FL > IR				
	**S(T22):** BA = FL = IR				
(h) Hepatocyte Lesion Index					
Site (S)	2	9819.800	141.814	**<0.001**	**0.826**
Time (T)	1	106.667	1.540	0.220	0.002
S × T	2	28.067	0.405	0.669	0.000
Residuals	54	69.244			
Scott-Knott tests	**S:** BA > IR > FL				
(i) Fulton's K					
Site (S)	2	0.017	1.174	0.313	0.003
Time (T)	1	0.031	2.112	0.149	0.009
S × T	2	0.035	2.395	0.096	0.023
Residuals	109	0.015			
Scott-Knott tests	**S:** BA = FL = IR				
	**T:** 15 = 22				

	**df**	**Deviance**	***P***	
(j) Melano-macrophage centers				
Site (S)	2	157.836	**<0.001**	**0.677**
Time (T)	1	0.021	0.845	0.000
S × T	2	0.019	0.983	0.000
Residuals	54			
Least square means test	**S: BA = IR > FL**			

Table S2. Analyses of variance of biochemical biomarkers assessed from plasma of fish sampled at three distinct Sites (S): Barragem (BA), Floresta (FL) and Iratim (IR) after 15 months of exposure (Time, T15) and 22 months (Time, T22). Effect sizes measures (ω^2) are presented for each term of the ANOVA model. For Scott-Knott pairwise *a posteriori* comparisons: "<" and ">" indicate $P < 0.05$ and "=" indicates $P > 0.05$. Significant terms used in pairwise tests ($\alpha = 0.05$) and those that contributed to the largest proportion of total variance are highlighted in bold.

	**df**	**MS**	***F***	***P***	**ω^2**
(a) AST					
Site (S)	2	5916	390.188	**<0.001**	**0.927**
Time (T)	1	48	3.182	0.080	0.003
S × T	2	5	0.336	0.7162	0.000
Residuals	54	15			
Scott-Knott tests	**S:** BA > IR > FL				
(b) ALT (log)					
Site (S)	2	2.177	323.212	**<0.001**	**0.915**
Time (T)	1	0.016	2.297	0.135	0.002
S × T	2	0.002	0.245	0.783	0.000
Residuals	54	0.007			
Scott-Knott tests	**S:** BA > IR > FL				
(c) AST/ALT ratio					
Site (S)	2	1.312	11.010	**<0.001**	**0.258**
Time (T)	1	0.009	0.079	0.779	0.000
S × T	2	0.024	0.200	0.819	0.000
Residuals	54	0.119			
Scott-Knott tests	**S:** FL > IR > BA				
(d) LDH (log)					
Site (S)	2	0.664	65.804	**<0.001**	**0.694**
Time (T)	1	0.001	0.076	0.784	0.000
S × T	2	0.000	0.023	0.977	0.000

Residuals	54	0.010			
Scott-Knott tests	**S:** BA > IR > FL				
(e)ALT/LDH ratio					
Site (S)	2	0.001	27.808	**<0.001**	**0.481**
Time (T)	1	0.000	0.612	0.437	0.000
S × T	2	0.000	0.130	0.878	0.000
Residuals	54	0.000			
Scott-Knott tests	**S:** BA = IR > FL				
(f) ALP (log)					
Site (S)	2	0.606	50.398	**<0.001**	**0.623**
Time (T)	1	0.008	0.668	0.417	0.000
S × T	2	0.013	1.095	0.342	0.001
Residuals	54	0.012			
Scott-Knott tests	**S:** BA > IR > FL				
(g) GamaGT					
Site (S)	2	740.3	116.696	**<0.001**	**0.790**
Time (T)	1	17.3	2.724	0.105	0.006
S × T	2	5.4	0.853	0.432	0.000
Residuals	54	6.3			
Scott-Knott tests	**S:** BA > IR > FL				
(h) GGT/ALP ratio					
Site (S)	2	0.369	59.094	**<0.001**	**0.660**
Time (T)	1	0.006	1.022	0.317	0.000
S × T	2	0.006	0.904	0.411	0.000
Residuals	54	0.006			
Scott-Knott tests	**S:** BA = IR > FL				

(i) Albumin (log)					
Site (S)	2	1.255	102.666	**<0.001**	**0.779**
Time (T)	1	0.045	3.663	0.061	0.010
S × T	2	0.001	0.067	0.935	0.000
Residuals	54	0.012			
Scott-Knott tests	**S:** FL > IR > BA				
(j) Total Protein					
Site (S)	2	22.170	57.148	**<0.001**	**0.643**
Time (T)	1	1.908	4.919	**0.031**	0.022
S × T	2	0.066	0.171	0.844	0.000
Residuals	54				
Scott-Knott tests	**S:** BA > IR > FL				
	**T:** 22 > 15				
(k) Globulin					
Site (S)	2	42.420	108.518	**<0.001**	**0.770**
Time (T)	1	2.760	7.052	**0.0104**	0.022
S × T	2	0.060	0.144	0.8659	0.000
Residuals	54	0.390			
Scott-Knott tests	**S:** BA > IR > FL				
	**T:** 22 > 15				
(l) Albumin/Globulin ratio (√)					
Site (S)	2	0.431	86.537	**<0.001**	**0.735**
Time (T)	1	0.022	4.440	**0.040**	0.015
S × T	2	0.001	0.164	0.8494	0.000
Residuals	54	0.005			
Scott-Knott tests	**S:** FL > IR = BA				
	**T:** 15 > 22				

Table S3. Organic pollutants detect in water, sediment and muscle of fish sampled at Barragem (BA), Floresta (FL) and Iratim (IR) sites. Muscle samples were obtained at 15 months and 22 months of exposure to reservoir waters. Bold values indicate concentrations exceeding the reference values established by Brazilian legislation [50].

	**Water**			**Sediment**			**Muscle**					
							15 months			*22 months*		
	**BA**	**FL**	**IR**	**BA**	**FL**	**IR**	**BA**	**FL**	**IR**	**BA**	**FL**	**IR**
∑PCBs	0.08	0.94	0.09	0.20	0.03	0.12	1.52	1.74	2.98	1.92	2.41	2.40
∑ 7PCBs	0.08	0.68	0.09	< DL	< DL	< DL	0.26	0.32	0.58	<DL	0.58	0.87
PCB 33							0.45	0.35	0.58	0.39	0.39	0.28
PCB 49							0.42	0.35	0.56	0.41	0.55	0.60
PCB 74							0.39	0.72	1.26	0.75	0.89	0.65
PCB 153							0.26	0.32	0.58	<DL	0.58	0.60
HBC	< DL	< DL	< DL	< DL	< DL	< DL						
∑HCHs	< DL	< DL	< DL	< DL	< DL	< DL						
∑Heptachlor	< DL	< DL	< DL	< DL	< DL	< DL						
∑Drins	< DL	< DL	< DL	< DL	< DL	< DL						
∑Chlordanes	< DL	< DL	< DL	< DL	0.04	< DL						
∑DDTs (DDE + DDD _+ DDT)	< DL	1.39	0.45	0.28	< DL	< DL	< DL	< DL	< DL	< DL	< DL	< DL

Endosulfan	< DL	< DL	< DL	< DL	< DL	< DL						
Methoxychlor	< DL	< DL	< DL	< DL	< DL	< DL						
Mirex	< DL	< DL	< DL	< DL	< DL	< DL						
∑ PBDEs	< DL	< DL	< DL	< DL	< DL	< DL	< DL	< DL	< DL	< DL	< DL	< DL
Naphthalene				4.11	3.56	9.08	6.65	7.28	7.67	8.21	7.70	32.2
Biphenyl				< DL	9.85	8.91						
Acenaphthylene				< DL	1.52	0.70						
Phenanthrene				0.63	**75.1**	4.85	6.51	< DL	6.70	6.60	< DL	7.83
Anthracene				< DL	6.91	0.72						
Fluoranthene				< DL	**115.1**	2.79	< DL	7.29	8.36	<DL	6.66	32.2
Pyrene				0.88	**73.0**	2.90	9.11	16.9	17.2	10.5	16.9	27.1
Benzo[a]anthracene				< DL	**39.0**	1.44						
Chrysene				< DL	37.3	2.16						
Benzo[b,j,k]fluoranthene				0.61	59.4	1.98						
Benzo[e]pyrene				< DL	21.7	0.91						
Benzo[a]pyrene				< DL	6.41	0.60						
C1-naphthalene				0.68	**26.6**	5.96						
C2- naphthalene				3.79	28.3	11.9						
C3- naphthalene				1.75	12.4	11.5						

C1-fluorene				0.61	8.08	1.39						
C1-phenanthrene				1.61	26.2	5.09	23.0	14.9	28.3	25.4	15.9	23.5
C2- phenanthrene				1.97	8.72	4.29						
C1-fluoranthene				0.58	22.4	1.80						
C1-pyrene				< DL	3.60	1.45	< DL	9.57	7.34	<DL	10.4	6.24
C1-chrysene				0.64	20.9	1.92						
∑PAHs	< DL	0.025	< DL		709.3	83.0	45.3	539.5	92.0	50.7	57.6	138.8
∑16 EPA PAHs	< DL	0.025	< DL		630.0	32.1	22.3	435.5	39.9	25.3	31.3	80.3
Alkyl PAHs					157.2	45.3	23.0	24.5	42.4	25.4	26.3	58.5
PAHs (2-3 rings)					6.91	0.72	13.2	7.28	14.4	14.8	7.7	40
PAHs (4-6 rings)					409.3	12.8	9.11	507.7	35.2	10.5	23.6	40.3

Table S4. Trace elements detect in water and sediment sampled at Barragem (BA), Floresta (FL) and Iratim (IR) sites. Bold values indicate concentrations exceeding the reference values established by Brazilian legislation [50].

	**Water (ug/l)**			**Sediment (ug/g)**		
	**BA**	**FL**	**IR**	**BA**	**FL**	**IR**
Al	59.6	**125**	**123**	35 ± 3	37 ± 3	22 ± 2
Cd	**11.8**	**12.1**	<QL	<QL	<QL	<QL
Cu	<QL	<QL	<QL	0.39 ± 0.01	0.34 ± 0.01	0.30 ± 0.01
Fe	210	**431**	280	76 ± 6	66 ± 4	57 ± 6

Mn	31.5	50.7	17.9	1.3 ± 0	0.6 ± 0	1.1 ± 0
Zn	13.5	15.9	21.6	0.12 ± 0.01	0.12 ± 0.01	0.15 ± 0.01
As	<QL	8.14	<QL	**33.1 ± 2.3**	**40.6 ± 2.8**	**20.5 ± 1.9**
Pb	<QL	<QL	<QL	24.2 ± 0.8	18.5 ± 1.5	20.5 ± 31.3
